# Early Developmental Zebrafish Embryo Extract to Modulate Senescence in Multisource Human Mesenchymal Stem Cells

**DOI:** 10.3390/ijms20112646

**Published:** 2019-05-29

**Authors:** Federica Facchin, Francesco Alviano, Silvia Canaider, Eva Bianconi, Martina Rossi, Laura Bonsi, Raffaella Casadei, Pier Mario Biava, Carlo Ventura

**Affiliations:** 1 Department of Experimental, Diagnostic and Specialty Medicine (DIMES), University of Bologna, Via Massarenti 9, 40138 Bologna, Italy; federica.facchin2@unibo.it (F.F.); francesco.alviano@unibo.it (F.A.); eva.bianconi2@unibo.it (E.B.); martina.rossi12@unibo.it (M.R.); laura.bonsi@unibo.it (L.B.); carlo.ventura@unibo.it (C.V.); 2 National Laboratory of Molecular Biology and Stem Cell Bioengineering of the National Institute of Biostructures and Biosystems (NIBB)–Eldor Lab, at the Innovation Accelerator, CNR, Via Piero Gobetti 101, 40129 Bologna, Italy; 3 Department for Life Quality Studies (QuVi), University of Bologna, Corso D’Augusto 237, 47921 Rimini, Italy; r.casadei@unibo.it; 4 Scientific Institute of Research and Care Multimedica, Via Milanese 300, 20099 Sesto San Giovanni (Milano), Italy; biava@tiscali.it

**Keywords:** stem cells, senescence, zebrafish embryo extract, senescence-associated β-galactosidase activity, adipogenesis, *TERT*, *BMI1*, *p53*, *p21*, *p16*

## Abstract

Stem cells undergo senescence both in vivo, contributing to the progressive decline in self-healing mechanisms, and in vitro during prolonged expansion. Here, we show that an early developmental zebrafish embryo extract (ZF1) could act as a modulator of senescence in human mesenchymal stem cells (hMSCs) isolated from both adult tissues, including adipose tissue (hASCs), bone marrow (hBM-MSCs), dental pulp (hDP-MSCs), and a perinatal tissue such as the Wharton’s Jelly (hWJ-MSCs). In all the investigated hMSCs, ZF1 decreased senescence-associated β-galactosidase (SA β-gal) activity and enhanced the transcription of *TERT*, encoding the catalytic telomerase core. In addition, it was associated, only in hASCs, with a transcriptional induction of *BMI1*, a pleiotropic repressor of senescence. In hBM-MSCs, hDP-MSCs, and hWJ-MSCs, *TERT* over-expression was concomitant with a down-regulation of two repressors of *TERT*, *TP53* (*p53*), and *CDKN1A* (*p21*). Furthermore, ZF1 increased the natural ability of hASCs to perform adipogenesis. These results indicate the chance of using ZF1 to modulate stem cell senescence in a source-related manner, to be potentially used as a tool to affect stem cell senescence in vitro. In addition, its anti-senescence action could also set the basis for future in vivo approaches promoting tissue rejuvenation bypassing stem cell transplantation.

## 1. Introduction

The human body continuously relies upon its own tissue-resident stem cells to repair adult tissues and organs, and to oppose senescence-related processes [1,2,3,4,5,6]. Human mesenchymal stem cells (hMSCs) exhibit self-renewal, multilineage differentiation and can be isolated and expanded in vitro from virtually all organs, even if bone marrow and subcutaneous fat are the elected sources [7]. Furthermore, hMSCs can be isolated not only from adult tissues but also from several fetal and perinatal sources [8].

As a result, in the last decade several attempts have been made to unfold hMSC features into regenerative medicine approaches for the treatment of a variety of tissue injuries and degenerative disorders [9,10,11,12,13,14,15]. Within this context, the use of synthetic and natural molecules, as well as electromagnetic fields and mechanical vibrations, can largely contribute to the development of strategies that may counteract stem cell senescence allowing the preservation of tissue homeostasis and our innate self-healing potential [16].

In particular, in neurodegenerative diseases, strategies that prevent telomere loss or increase telomere length in MSCs, as well as the use of neuropeptides that elicit the regulation of the Wnt/β-catenin signaling pathway (involved in the shift of MSCs towards a senescent phenotype), may prevent the symptoms of neurodegenerative disorders and improve the results of MSCs-based therapy [17]. At the same time, therapeutic approaches of osteoarthritis are based on strategies able to stimulate MSCs through telomerase activators, mechanical strain, and epigenetic regulation in order to maintain their chondrogenic differentiation potential and to counteract homeostasis alterations occurring in cell in vitro cultivation and expansion [18].

Another possible and useful way to resist stem cell senescence is mimicking organisms that have developed a robust ability to regenerate tissues. Zebrafish (*Danio rerio*), for example, is widely used as an animal model to study regeneration and organogenesis, given its ability to regenerate organs, such as the heart or the central nervous system, at a noticeable higher efficacy than in humans [19,20,21,22,23]. Despite the evolutionary distance between humans and zebrafish, hMSCs can still perceive ancestral microenvironmental cues from this species [24] and several scientific researches in comparative biology revealed interesting conserved evolutionary patterns in tissue regeneration.

Previously, we described a putative anti-senescence action of a zebrafish extract (ZF1) which was obtained from embryos at an early stage of development (50% epiboly) in hMSCs that were isolated from the adipose tissue (human adipose tissue-derived stem cells, hASCs) and cultured from the third to the 5th passage, in order to provide a preliminary investigation of its effects on cell viability, stemness, and senescence regulatory patterning [25]. In particular, ZF1 which was given at a concentration of 10 μg/mL for 72 h did not influence cell viability or apoptosis, while it enhanced the transcription of the *telomerase reverse transcriptase* (*TERT*), *BMI1 proto-oncogene, polycomb ring finger* (*BMI1*), and the stemness *POU domain class 5 homeobox 1 (POU5F1)* (alias *Oct-4*), *Sox-2,* and *v-myc avian myelocytomatosis viral oncogene homolog* (*c-Myc*) genes [25].

Recently, we investigated the role of ZF1 at the same dose and for the same exposure time on hASCs at four different, considerably more prolonged, subculture stages (5th, 10th, 15th and 20th) in order to assess additional biological responses to the treatment with ZF1. Our results showed that ZF1 is a feasible tool to modulate and reverse hASC senescence in long-term culturing conditions [26].

Since the zebrafish is considered one of the most popular vertebrate models in developmental biology and biomedical research, several transcriptomic and proteomic analyses have been increasingly used for profiling important molecules of zebrafish at different developmental stages or in specific organs/tissues or experimental conditions [27,28,29,30,31,32,33,34,35,36,37,38,39,40].

Only one study [36], among all considered, reported the transcriptome at the specific developmental stage of our interest (50% epiboly) and a second reported a list of proteins isolated from the ZF1 extract [41].

In the present study, we first explored the anti-senescence role of ZF1 in hASCs treated for 72 h with ZF1 at five different concentrations (0.01, 10, 20, 40, and 100 μg/mL), in order to evaluate the eligible ZF1 dose to use in the present work.

Then, we investigated whether the anti-senescence action of ZF1 at a defined concentration can be considered a general feature applicable to various types of hMSCs isolated from adult and perinatal tissues.

Therefore, we investigated cell proliferation, senescence-associated β-galactosidase (SA β-gal) activity, adipogenic ability, gene transcription of *TERT*, encoding the catalytic core of telomerase, *BMI1*, a transcriptional regulator acting as a major repressor of senescence, *tumor protein p53* (*TP53,* alias *p53*), *cyclin dependent kinase inhibitor 1A* (*CDKN1A,* alias *p21*), *cyclin dependent kinase inhibitor 2A* (*CDKN2A,* alias *p16*), p21 protein expression in ZF1-treated hMSCs from adipose tissue (hASCs), bone marrow (hBM-MSCs), dental pulp (hDP-MSCs), and Wharton’s Jelly (hWJ-MSCs).

## 2. Results

### 2.1. hMSC Isolation and Phenotype Characterization

**Figure 1:**
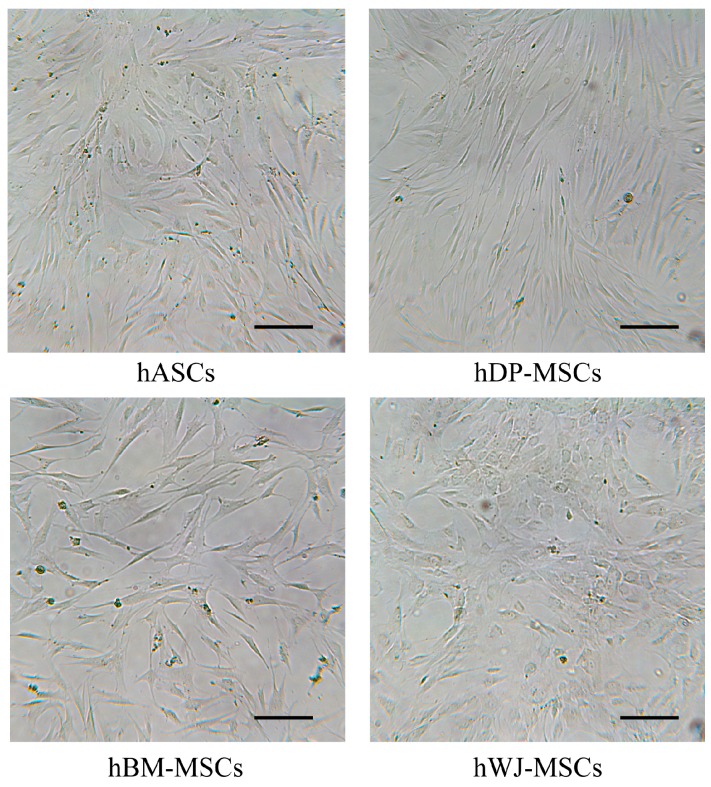
Representative images of hMSCs (culture passages 5th–7th) isolated from human adipose tissue (hASCs), dental pulp (hDP-MSCs), bone marrow (hBM-MSCs), and Wharton’s Jelly (hWJ-MSCs). Cells were analyzed for morphology under optical microscopy (40× magnification and bright field illumination). Scale bar corresponds to 200 μm.

Cells were isolated from different sources, as described in the Material and Methods section, and were selected for their ability to adhere to plastic surfaces. After two to three weeks of in vitro culture, cell populations showed the typical fibroblast-like morphology (Figure 1) and displayed an immune phenotype consistent with multipotent mesenchymal stem cells. In fact, cell populations were positive for markers CD29, CD44, CD73, CD105, and CD166; and negative for CD14, CD34, and CD45. These results were previously described in detail [42,43].

### 2.2. Evaluation of Different ZF1 Concentrations Cytotoxicity in hASCs

**Figure 2:**
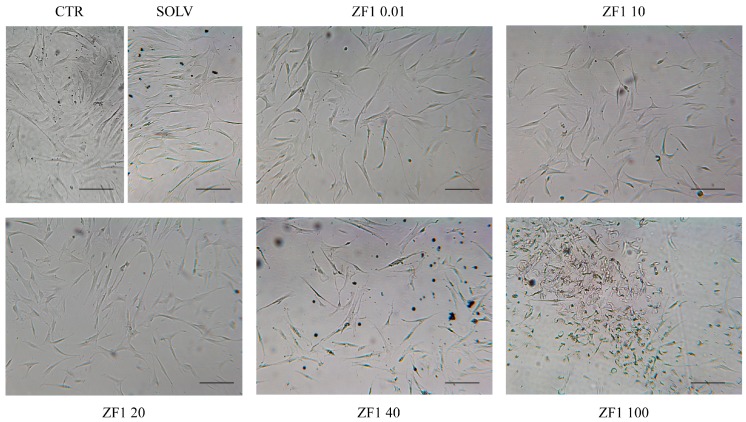
Representative images of hASCs treated with ZF1 at different concentrations (0.01, 10, 20, 40, and 100 μg/mL) or with a solvent (SOLV) or untreated (CTR). Cytotoxicity was evaluated with a morphological analysis. Images were obtained with an optical microscopy (40× magnification and bright field illumination). Scale bar corresponds to 200 μm.

A dose-response morphological analysis of the effects elicited by ZF1 on hASCs showed that the higher concentrations (40 and 100 μg/mL) which were investigated modified cell morphology and induced high mortality. This suggested a toxicity of the treatments, and consequently, lead to their exclusion from further experiments. In contrast, treatment with a solvent or with ZF1 at 0.01, 10, and 20 μg/mL did not alter the cellular morphology, as compared with control cells from the same culture passages (5th–7th) (Figure 2).

### 2.3. ZF1 Does Not Affect Proliferation in hASCs

**Figure 3:**
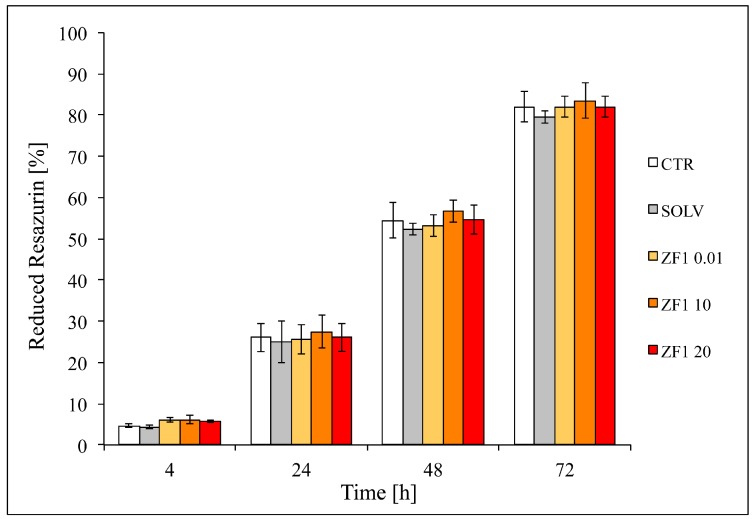
Effects of different concentrations of ZF1 on metabolic activity in hASCs. The percentage of resazurin reduction (as an indicator of cell proliferation) in hASCs treated with ZF1 (0.01, 10, and 20 μg/mL) or a solvent (SOLV) or untreated (CTR). An analysis was conducted at 4, 24, 48, and 72 h from the beginning of the treatment and an experiment was performed in technical quadruplicate. Statistical significance was evaluated using ANOVA followed by the Tukey HSD test. Data are expressed as mean ± standard deviation (SD), *n* = 4.

The preliminary in vitro toxicology resazurin-based assay performed on hASCs in technical quadruplicate showed a comparable proliferation rate in all the tested experimental conditions (control, solvent, and ZF1 at 0.01, 10, and 20 μg/mL), indicating that treatments had a nontoxic effect and that ZF1 did not affect hASC proliferation (Figure 3).

On the basis of these results, we decided to use a solvent as a control for subsequent experiments.

### 2.4. ZF1 Decreases SA β-Gal Staining and Increases TERT Gene Expression in hASCs

**Figure 4:**
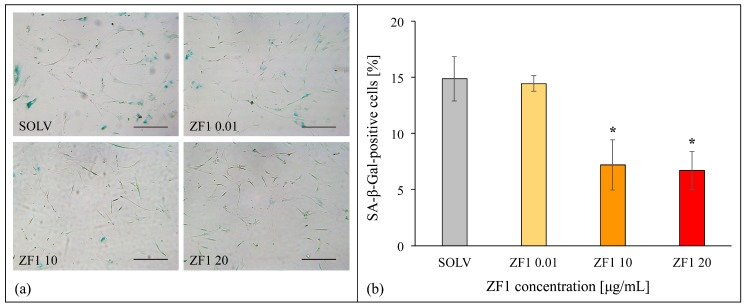
Effects of different concentrations of ZF1 on SA β-gal activity in hASCs. The hASCs (culture passages 5th–7th) were seeded in 6-well plates and were cultured in the presence of 0.01, 10, and 20 μg/mL ZF1, or a solvent as a control for 72 h, then processed for SA β-gal assessment. (**a**) Images represent hASCs after SA β-gal staining. SA β-gal positive cells are blue. The scale bar corresponds to 200 μm; (**b**) Positive (blue) and negative (not colored) cells were counted in at least three random fields for each technical replicate under the microscope (200× magnification and bright field illumination). Data represent the percentage of SA β-gal positive cells calculated as the number of positive cells divided by the total number of counted cells multiplied by 100 (percentage of blue cells ± SD, *n* = 3, statistical significance was calculated using the Student’s *t*-test, * *p* < 0.05).

A dose-response analysis of the effects elicited by ZF1 on SA β-gal staining was set up in hASCs to identify the most effective concentration influencing the expression of this senescence marker. Cells (culture passages 5th–7th) were treated for 72 h with ZF1 at the final concentrations of 0.01, 10, and 20 μg/mL. Although 0.01 μg/mL ZF1 was ineffective, both 10 and 20 μg/mL ZF1 significantly reduced the number of senescent hASCs positively blue stained for SA β-gal (*p* < 0.05) (Figure 4).

**Figure 5:**
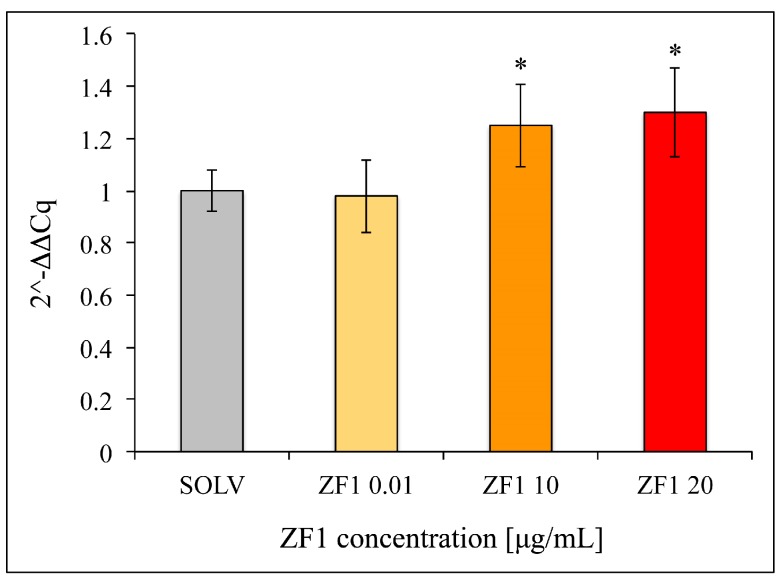
The effect of ZF1 treatment on *TERT* gene expression in hASCs. The hASCs (culture passages 5th–7th) were exposed for 72 h in the presence of 0.01, 10, and 20 μg/mL ZF1, or solvent (SOLV) as a control. The expression value of the transcripts evaluated in solvent or ZF1-treated cells was normalized to the expression levels of three reference genes, *HPRT1*, *GAPDH* and *TBP*. The amount of the target mRNA in the ZF1 treated cells was plotted as the fold change over the expression in control cells. The experiment was performed in technical triplicate. Statistical significance was evaluated by using the CFX Manager Software version 3.1 (Bio-Rad Laboratories) and Student’s t-test (mean ± SD, *n* = 3, * *p* < 0.05).

Consistent with the experiments assessing the effect of ZF1 on SA β-gal activity, hASCs (isolated from one subject) and treated with ZF1 at 0.01 μg/mL concentration showed a gene expression value of the catalytic subunit of telomerase (*TERT*) similar to that of control cells (SOLV). In contrast, hASCs exposed to both 10 μg/mL and 20 μg/mL ZF1 resulted in a similar statistically significant increase in *TERT* transcription as compared with the control hASCs (SOLV) (Figure 5).

### 2.5. ZF1 Promotes Adipogenesis in hASCs

**Figure 6:**
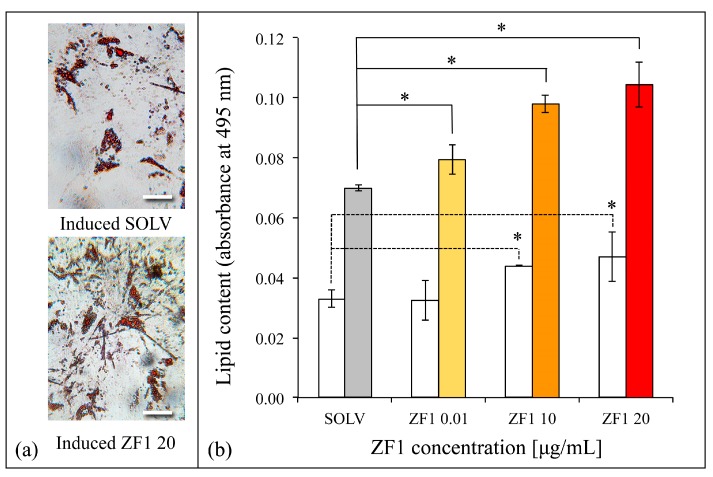
Effects of ZF1 treatment on adipogenic differentiation in hASCs at different concentrations. The hASCs (culture passages 5th–7th) were seeded in 24-well plates and were cultured in the presence of 0.01, 10, and 20 μg/mL ZF1, or solvent (SOLV) as a control for 72 h. (**a**) Images represent hASCs Oil red O staining after treatment with solvent (**above**) or ZF1 20 μg/mL (**below**) and adipogenic medium. Cells positive for adipogenesis showed red colored vacuoles in cytoplasm. Scale bar corresponds to 100 μm; (**b**) White histograms represent data derived from hASCs cultured in basal medium, while colored histograms represent those from hASCs treated with adipogenic medium. The lipid-rich vacuoles Oil Red O dye was extracted by wells and its absorbance was read at 495 nm with a spectrophotometer. Data are expressed as mean of lipid content at 495 nm absorbance ± SD. Horizontal dashed or continuous black lines represent the significance of differences between data obtained from hASCs cultured in basal and from an adipogenic medium, respectively (statistical significance was calculated using the Student’s *t*-test, * *p* < 0.05, *n* = 3).

To better investigate the effect of ZF1 on hASCs, adipogenic differentiation after 0.01, 10, and 20 μg/mL treatment was evaluated and quantified via Oil Red O staining, a neutral triglycerides and lipids dye. During differentiation, the hASCs produce multiple lipid-rich vacuoles in the cytoplasm, which increased in their size and number during the two weeks of induction, and they showed an intense red color if stained with Oil Red O (Figure 6a). The red staining quantification revealed that ZF1 enhanced hASC adipogenic commitment both when cells grew in a culture medium and when cells were induced. Moreover, the statistically significant effect was dose-dependent (Figure 6b).

Therefore, based on the above results obtained with hASCs, we decided to use ZF1 at 20 μg/mL in the following experiments performed on all the four selected hMSC types.

### 2.6. ZF1 and Modulation of Cell Proliferation in hMSCs Isolated from Four Different Sources

**Figure 7:**
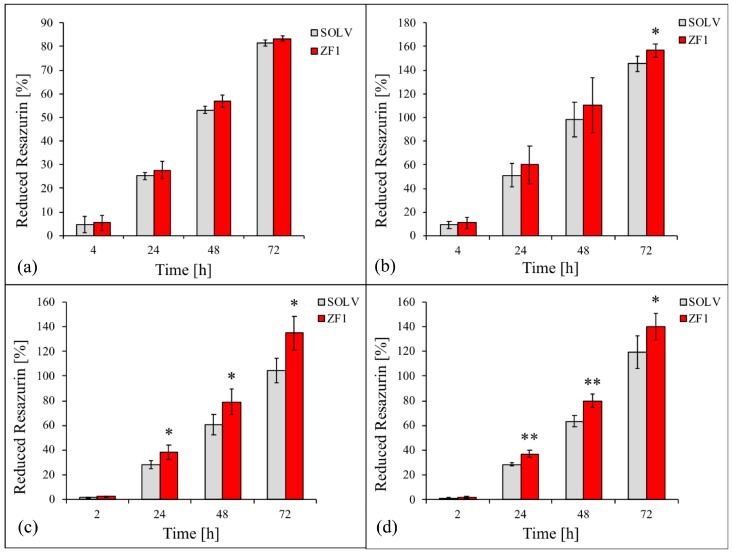
The effects of different concentrations of ZF1 on metabolic activity in hMSCs. The hASCs (**a**), hBM-MSCs (**b**), hDP-MSCs (**c**) and hWJ-MSCs (**d**), at passages 5th–7th, were exposed for 72 h in the presence of 20 μg/mL ZF1, or solvent as a control (SOLV). An analysis of the percentage of the reduction of resazurin (as an indicator of cell proliferation) was conducted at 4, 24, 48, and 72 h from the beginning of the treatment and data are expressed as mean (*n* = 3) ± SD. Statistical significance was analyzed with ANOVA followed by the Tukey HSD test, * *p* < 0.05, ** *p* < 0.01.

The adult stem cells, hASCs, hDP-MSCs, and hBM-MSCs, and perinatal stem cells, hWJ-MSCs, (all at culture passages 5th–7th) were treated for 72 h with 20 μg/mL ZF1 or with its solvent as a control. As shown in Figure 7a, the percentage of reduction of resazurin (as an indicator of cell proliferation) did not change in the ZF1 treated hASCs as compared with solvent treated cells at any experimental time point, in accordance with our previous assay results reported above.

In contrast, in hBM-MSCs, hDP-MSCs, and hWJ-MSCs cell proliferation increased after ZF1 treatment, reaching statistical significance at different experimental points: at 72 h for all three cell types (*p* < 0.05) and also at 24 h and 48 h for hDP-MSCs and hWJ-MSCs (Figure 7b–d).

### 2.7. ZF1 Reduces SA β-Gal Staining in hMSCs Isolated from Four Different Sources

**Figure 8:**
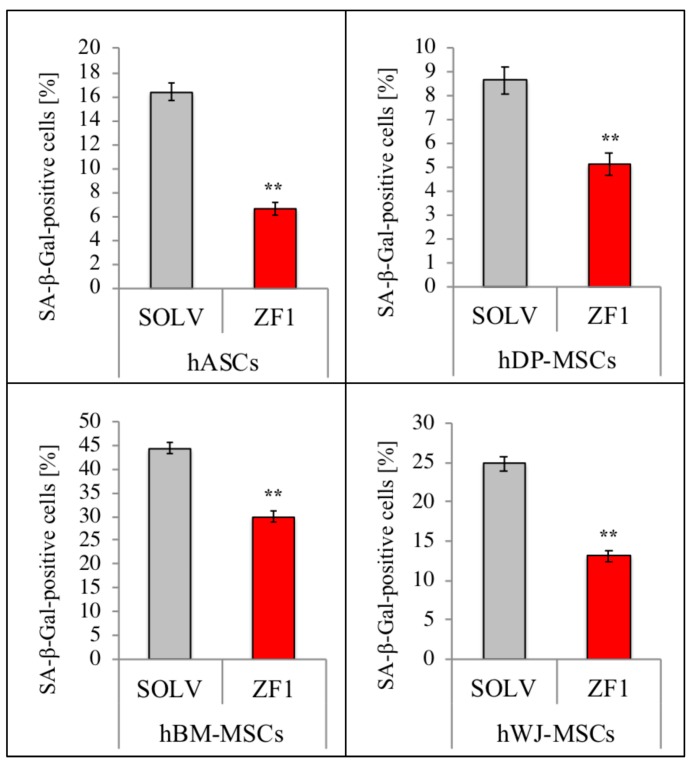
ZF1 counteracts SA β-gal activity in hMSCs. The hMSCs (culture passages 5th–7th) were cultured for 72 h in the presence of 20 μg/mL ZF1, or a solvent as a control (SOLV). Then, cells were processed for SA β-gal assessment, as described in Figure 4 legend. The experiments with hASCs, hDP-MSCs, hBM-MSCs, and hWJ-MSCs were all performed with cells derived from three subjects. Data represent the percentage of SA β-gal positive cells calculated as the number of positive cells divided by the total number of counted cells multiplied by 100. All ZF1 treated cells were significantly different from the control group (Proportion in % ± SD, *n* = 3). Statistical significance was evaluated using the Z-test for proportional, ** *p* < 0.01.

**Figure 9:**
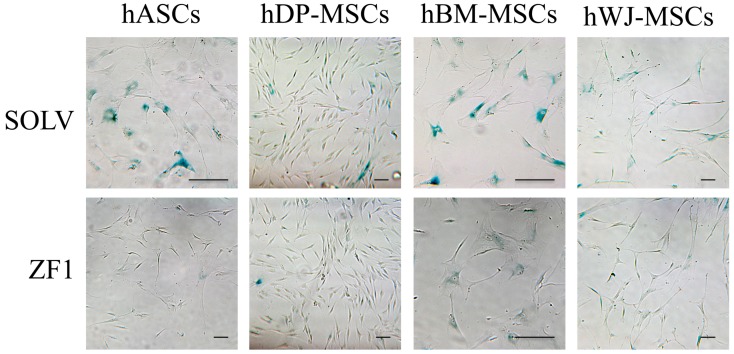
Representative images of the effects of ZF1 treatment on SA β-gal activity in hMSCs. The hMSCs from different sources (culture passages 5th–7th) were treated with 20 μg/mL ZF1 or with a solvent (SOLV) as a control, and then analyzed for SA β-gal expression (blue color, 200× magnification and bright field illumination). Images are representative of three separate experiments (*n* = 3) for each hMSC type.

All investigated hMSCs (culture passages 5th–7th) were treated for 72 h with 20 μg/mL ZF1 or with its solvent as a control and tested with the SA β-gal staining assay. As shown in Figure 8, the number of senescent stem cells expressing SA β-gal was remarkably reduced by the treatment, as compared with the control cells, in each investigated mesenchymal lineage (*n* = 3, *p* < 0.01). The different distribution of SA β-gal positive cells among ZF1-exposed and untreated hMSCs of each source is also evident in Figure 9, showing the relevant decline of senescent marker expression in ZF1 treated cells. Additionally, Figure 9 shows that the treatment with ZF1 at the investigated concentration did not alter the cellular morphology, as compared with the control cells, as previously discussed in Section 2.2.

### 2.8. ZF1 and Transcriptional Modulation of Stem Cell Senescence in Multisource hMSCs

**Figure 10:**
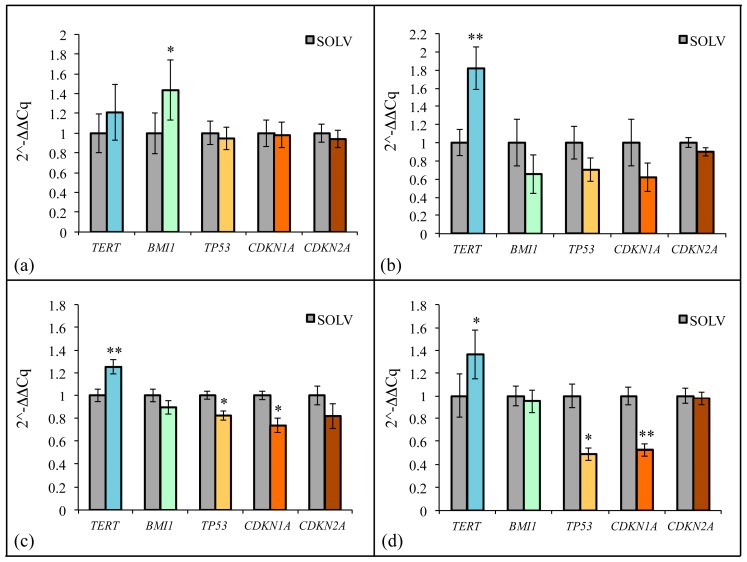
Effect of ZF1 on gene expression in hMSCs. The hASCs (**a**), hBM-MSCs (**b**), hDP-MSCs (**c**) or hWJ-MSCs (**d**), at passages 5th–7th, were exposed for 72 h in the presence of 20 μg/mL ZF1, or a solvent (SOLV) as a control. The expression value of the transcripts evaluated in the solvent or ZF1-treated cells, was normalized to the expression levels of three reference genes, *HPRT1*, *GAPDH,* and *TBP*. The amount of the target mRNA in the ZF1 treated cells was plotted as the fold change over the expression in the control cells. The experiments were all performed with cells derived from three subjects. Statistical significance was evaluated by using the CFX Manager Software version 3.1 (Bio-Rad Laboratories) and the Student’s *t*-test (mean ± SD, * *p* < 0.05, ** *p* < 0.01, *n* = 3).

The relative quantitative real-time PCR (qPCR) analysis revealed that hASCs, hBM-MSCs, hDP-MSCs, and hWJ-MSCs (culture passages 5th–7th) all responded to a 72-h treatment with 20 μg/mL ZF1 with an evident increase in *TERT* gene transcription as compared with the ZF1 unexposed (control) cells (Figure 10). Next, we investigated whether ZF1 may have elicited its anti-senescence effect by acting on the transcription of other senescence modulators. In Figure 10a, we show that, in hASCs, ZF1 was also able to enhance the gene expression of *BMI1*, a pleiotropic transcriptional regulator acting as a major repressor of senescence [44,45,46], while the exposure to ZF1 of hBM-MSCs, hDP-MSCs or hWJ-MSCs resulted in a nonsignificant effect on *BMI1* transcription (Figure 10b, 10c, and 10d). Moreover, in hASCs, the treatment with ZF1 failed to affect the transcription of *TP53 (p53)*, as well as the gene expression of type 1A and 2A cyclin dependent kinase inhibitors, *CDKN1A (p21)* and *CDKN2A (p16)* (Figure 10a). On the contrary, in hBM-MSCs, hDP-MSCs, and hWJ-MSCs, the ZF-induced increase in *TERT* transcription was associated with a down-regulation in both *TP53 (p53)* and *CDKN1A (p21)* transcription, with no significant changes in *CDKN2A (p16)* gene expression (Figure 10b–d, respectively).

### 2.9. ZF1 and p21 Expression in Multisource hMSCs

**Figure 11:**
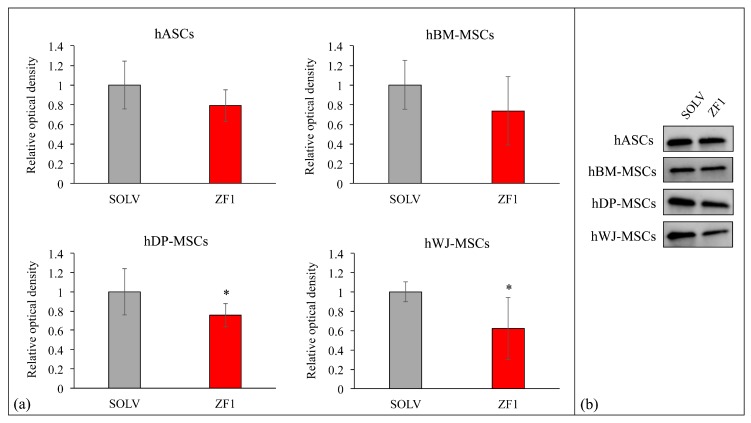
The Western blot analysis of p21 expression in SOLV- (as a control) and ZF1-treated hMSCs. (**a**) The graphs show the comparison of relative densitometric values of the bands normalized to total protein detected in stain-free acquisition in control (mean ± SD, statistical significance was evaluated using the Student’s *t*-test, * *p* < 0.05, *n* = 3). (**b**) Representative images of the Western blot for each investigated cell type.

Consistent with the experiments assessing the effect of ZF1 on gene transcription, we decided to perform a protein investigation for p21, since it is the last protagonist of the TERT/p53/p21 pathway. Western blot analysis revealed that a 72-h treatment with 20 μg/mL ZF1 affected the expression of p21 protein in the investigated hMSCs (culture passages 5th–7th), as compared with the ZF1 unexposed (control) cells (Figure 11). In particular, we showed that in hASCs and in hBM-MSCs, the change of p21 expression was not significant, while the exposure of hDP-MSCs or hWJ-MSCs to ZF1 resulted in a statistically significant decrease of the protein, in accordance with the relative *p21* gene transcription (Figure 11).

### 2.10. ZF1 and Adipogenic Commitment in hMSCs

**Figure 12:**
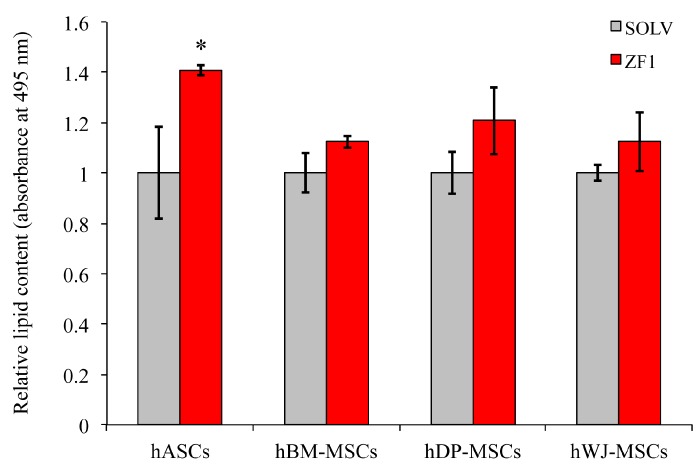
Effects of ZF1 treatment on adipogenic differentiation in hMSCs. The hASCs, hBM-MSCs, hDP-MSCs, and hWJ-MSCs, at the 5th passage, were exposed for 72 h in the presence of 20 μg/mL ZF1, or a solvent as a control (SOLV). An experiment was performed in technical triplicate for each cell type. At the end of treatment, hMSCs were incubated with adipogenic medium for two weeks and then differentiation was assessed using Oil Red O staining. The lipid-rich vacuoles Oil Red O dye was extracted from each well and its absorbance was read at 495 nm with a spectrophotometer. Data are expressed as mean relative lipid content (absorbance at 495 nm) of the ZF1 treated cells over the solvent treated ones (used as control, SOLV = 1) ± SD and statistical significance was evaluated using the Student’s *t*-test, * *p* < 0.05, *n* = 3.

The study of the role of ZF1 in hASC adipogenesis, described above, prompted us to extend our investigation to the other three hMSC types (hDP-MSCs, hBM-MSCs, and hWJ-MSCs), performed in technical triplicate. The hMSC adipogenic commitment was evident when cells were induced by adipogenic medium, as compared with cells grown in a culture medium (data not shown). The 72-h treatment with 20 μg/mL ZF1 increased the hASC adipogenic ability (*p* < 0.05) as compared with the ZF1 unexposed (control) cells, as previously described in preliminary experiments. In contrast, in hDP-MSCs, hBM-MSCs, and hWJ-MSCs, the results revealed that the treatment with ZF1 did not increase adipogenesis with statistical significance (Figure 12).

## 3. Discussion

The hMSCs are virtually present in all human organs and have different regenerative potential among the various tissue districts [47]. The properties of self-maintenance, multilineage differentiation, and trophic signaling that are exhibited by hMSCs make them highly attractive candidates for cell therapy approaches, endowing great advantages and peculiarities. The stem cell population that has been first isolated and characterized is hBM-MSCs. The hASCs and hDP-MSCs are commonly harvested in minimally invasive contexts, being abundant and rapidly proliferating stem cell populations, respectively. The hWJ-MSCs are obtained from discarded tissues and retain high plasticity and remarkable immunomodulatory properties due to their embryological and developmental origin [48,49,50,51]. In addition, the hMSCs from either adult or perinatal origins are not burdened by ethical problems and are supposed to be safer than embryonic stem cells or induced pluripotent stem (iPS) cells in terms of tumorigenesis and genomic modifications [52,53].

In an effort to use hMSCs from different tissue sources as potential tools for cell therapy, these cells are subjected to prolonged in vitro expansion in order to increase their number, and conceivably, their regenerative effect, prior to transplantation [10].

However, despite their diversity [54,55], all types of hMSCs undergo replicative senescence when cultured in vitro, a major drawback from the original assumption that tissue regeneration is promoted in a cell number-dependent fashion.

Here, we first show that the use of a developmentally defined zebrafish embryo extracted (ZF1) at concentrations of 10 and 20 μg/mL did not alter cell morphology and cell proliferation in hASCs as evidence of nontoxicity. Furthermore, ZF1 was able to counteract the hASC expression of a well-established senescence marker (SA β-gal) and it caused a significant increase in both induced and spontaneous adipogenic commitment, reinforcing our previous observations about hASCs [25,26]. In addition, similar effects on the β-gal activity were evident in other hMSCs that were investigated such as hBM-MSCs, hDP-MSCs, and hWJ-MSCs when treated with ZF1 at the defined concentration, however, adipogenic commitment was not significantly influenced. In particular, the different tissue origin could affect the MSC differentiation ability after ZF1 treatment [47,56].

In addition, the observed enhancement of *TERT* expression induced by ZF1 in all the investigated hMSC populations suggests that the rescue of a telomerase-dependent pathway could represent a common mechanism for the anti-senescence action of this embryo extract in hMSCs, independent of the stem cell source. The over-expression of *TERT* in hASCs reinforced the previously observed results in long-term cultures [26]. In particular, *TERT* expression is required to support optimal telomerase activity to counteract progressive telomere shortening and senescence [57]. Nevertheless, the observation, only in hASCs, that ZF1 enhanced the expression of *BMI1*, and the complex telomerase-dependent and -independent functions exploited by this chromatin remodeler in senescence repression [58,59] suggests that the anti-senescence effect of ZF1 may also entail pleiotropic, which are still unexplored dynamics, depending on the MSC source. Such a hypothesis is further supported by the finding that different from hASCs, in hBM-MSCs, hDP-MSCs, and hWJ-MSCs the over-expression of *TERT* induced by ZF1 was associated with a down-regulation in *TP53 (p53)* transcription and in both the gene and protein CDKN1A (p21) expression. In these hMSC types the opposite transcriptional responses induced by ZF1 may converge to potentiate the anti-senescence response as it may be inferred by the fact that the loss of p53 function has been found to accelerate telomerase activity [60], and that senescent cells upregulate cell cycle inhibitors (such as p53 and p21) [61]. Therefore, the observation that p53-dependent transcriptional repression of *TERT* was mediated by *CDKN1A (p21)* in both normal and pathologic cells [62,63] indicates that the down-regulation of *TP53 (p53)* and *CDKN1A (p21),* concomitant with *TERT* over-expression, may be part of a delicate circuitry through which ZF1 could affect senescence in hMSCs in a source-dependent manner, since a similar pattern was not observed in hASCs in response to ZF1 treatment. The lack of an effect of ZF1 on *CDKN2A (p16)* transcription in all investigated hMSCs suggests that ZF1 action may not involve previously reported BMI1/p16/pRB pathways [58,59].

Compounding the complexity of the putative mechanisms underlying the speculated anti-senescence response elicited by ZF1 are recent observations that provide evidence for a novel function of TERT in stem cell activation, which is independent of telomerase activity, as shown by the promoting effect on hair growth by TERT over-expression in a mouse strain that is lacking the RNA component of telomerase [64]. In this regard, it has also been found that TERT has a role in activating stem cells which is mediated by the transcriptional activation of a developmental program converging on Myc and Wnt signaling [65]. Therefore, the ZF1-mediated increase in *TERT* transcription may also involve a pleiotropic activation of stem cells, counteracting their senescence in a manner which is independent of the synthesis of telomere repeats. Further studies are in progress to discriminate among these putative telomerase-dependent and -independent pathways in hMSC response to ZF1. In fact, to date, we only have evidence about the ZF1 effect on telomerase activity in a long-term culture of hASCs [26]. As a next step, we plan to further investigate telomerase activity in other hMSCs under different experimental conditions. Starting from the information currently available on the presence of defined transcripts and proteins in zebrafish at the developmental stage of 50% epiboly [36,41], we will attempt to identify putative conductors capable of recapitulating the biological program observed in the present study.

On the whole, our results suggest that the anti-senescence action elicited by ZF1 on multisource hMSCs can be unfolded into two major, non-mutually exclusive, biomedical implications. Concerning the first implication, antagonizing stem cell senescence during prolonged in vitro culture may allow stem cells expansion up to the number precisely required for a targeted regenerative setting. As far as the second implication is concerned, the anti-senescence action promoted in vitro by ZF1 could be conceivably deployed in vivo using the extract to target the stem cells where they are in all body tissues and afford a process of tissue rejuvenation without the need for stem cell transplantation.

In future studies we are committed to extending the characterization of ZF1 composition and action, and also to addressing the potential limits, which include alterations in genome stability and eventual tumorigenic drift. We also plan to investigate the ZF1 effect on senescence progression at late passages in multiple hMSC types, with particular emphasis on WJ-hMSCs. In fact, these perinatal hMSCs retain the chance for higher multilineage commitment than other adult hMSCs [66]. For this reason, WJ-hMSCs are particularly amenable for future off-the-shelf approaches of allogeneic cell therapy where cells are conceived for long-lasting banking (years) prior to transplantation, an issue that will require effective and precise control of senescence drift over the banking time.

## 4. Materials and Methods

### 4.1. Ethics Statement

All the tissue samples were obtained from subjects that gave their informed consent for inclusion before their participation in the study. The study was conducted in accordance with the Declaration of Helsinki, and the protocol was approved by the local ethical committees (CE) (S. Orsola-Malpighi University Hospital, project identification code: n.1645/2014, ref. 35/2014/U/Tess and Villalba Hospital, project identification code: 16076 of Bologna, Italy).

### 4.2. hMSCs Harvesting and Culture

hASCs Human subcutaneous adipose tissue samples (obtained from 3 subjects) were isolated from lipoaspiration procedures and processed by using the Lipogems device (PCT/IB2011/052204), as previously described [43]. A volume of 1.5 mL of Lipogems product containing hASCs was seeded in a T75 flask (Falcon BD, Bedford, MA, USA) precoated with human fibronectin (0.55 μg/cm^2^) (Sigma-Aldrich Co., St. Louis, MO, USA) and human collagen I–III (0.50 μg/cm^2^) (ABCell-Bio, Paris, France) and cultured in alfa-minimal essential medium (α-MEM, Carlo Erba Reagents, Milano, Italy) supplemented with 10% heat-inactivated fetal bovine serum (FBS) (Gibco, Waltham, MA, USA), 1% penicillin-streptomycin solution, 1% l-glutamine 200 mM (Carlo Erba Reagents).

hDP-MSCs Vital human molars were obtained from 3 adult subjects during routine dental extraction. Dental pulp tissue fragments were recovered and digested as previously described [67]. The hDP-MSCs, slipped down from the explants, were isolated and cultured in Dulbecco’s modified Eagle’s medium high glucose (DMEM, BioWhittaker Cambrex, Walkersville, MD, USA) in the presence of 10% FBS (Gibco) and antibiotics (100 U/mL penicillin, 100 μg/mL streptomycin and 0.25 μg/mL amphotericin B) (Carlo Erba Reagents).

hBM-MSCs Bone marrow was collected from 3 healthy adult volunteers and treated as previously described [68]. The isolated hBM-MSCs were plated at 1 × 10^6^/cm^2^ in culture flasks in DMEM high glucose (BioWhittaker Cambrex) supplemented with 20% FBS (Gibco) and antibiotics (200 U/mL penicillin, 200 μg/mL streptomycin) (Carlo Erba Reagents).

hWJ-MSCs Umbilical cords from 3 healthy donor mothers obtained from caesarean sections were rapidly transferred to the laboratory and treated as previously described [69]. Cord fragments with an approximate diameter of 2 mm were seeded onto the surface of a culture dish with DMEM low glucose (BioWhittaker Cambrex) supplemented with 10% FBS (Gibco), penicillin and streptomycin 200 μg/mL (Carlo Erba Reagents).

All investigated cell lines were incubated at 37 °C in a humidified atmosphere with 5% CO_2_. The non-adherent cells were removed, and the medium was changed subsequently every 2–4 days. Confluent cells were detached by treatment with trypsin-EDTA (Sigma-Aldrich Co.), maintained and expanded until the desired culture passages.

Before their experimental use, mesenchymal stem cells obtained from all investigated culture tissues at the same doubling passage were observed under the optical microscope (at 40× magnification and bright field illumination). Therefore, cells were analyzed with flow cytometry to confirm the presence of minimal criteria for defining multipotent mesenchymal stem cells, in accordance with the International Society for Cellular Therapy [8].

### 4.3. Zebrafish Embryo Extract, Dose Analysis and Treatments

Zebrafish embryos were harvested and processed as previously described [25,70]. The 50% epiboly (5 h and 15 min post-fertilization, hpf) developmental stage (named here ZF1) was chosen and the extract containing eggs at the density of 100/mL was prepared in a glycero-alcoholic solution (60% glycerol, 5% ethanol, 0.12% potassium sorbate and 0.08% sodium benzoate) and stored at 4 °C until use, according to the manufacturer’s standard protocol (Aurora Biosearch, Bollate, Milano, Italy). The ZF1 extract was previously analysed on a one-dimensional sodium dodecyl sulphate-polyacrylamide gel electrophoresis (SDS-PAGE) and its proteic content was characterized by using liquid chromatography–tandem mass spectrometry (LC-MS/MS) [41]. In order to evaluate the eligible ZF1 dose to use in the present work, hASCs were first treated for 72 h with ZF1 at five different concentrations (0.01, 10, 20, 40, and 100 μg/mL), analyzed under a light microscope (at 200× magnification and bright field illumination), and then submitted to preliminary analyses. On the basis of the results from these treatments, investigated hMSCs were cultured with 20 μg/mL ZF1 for 72 h in all experiments. An equal amount of ZF1 solvent (a glycero-alcoholic solution) was used as a control in all experiments except in the resazurin-based assay where both control and solvent were used.

### 4.4. BCA Protein Assay

The protein content of ZF1 was determined using a BCA protein assay kit, following the manufacturer’s instructions (Pierce Biotechnology, Rockford, IL, USA). As previously described, the protein content of the extract was determined using a Nanodrop instrument (Nanodrop ND 1000 v.3.8.1, Wilmington, DE, USA), with serial dilution of bovine serum albumin as a standard [25].

### 4.5. In Vitro Resazurin-Based Toxicology Assay

To evaluate the ZF1 cell proliferation as a function of metabolic activity of the cells, In Vitro Toxicology Assay kit, resazurin-based, (Sigma-Aldrich Co.) was preliminarily used on the hASCs at the 5th culture passage, as representative of the investigated mesenchymal cells. These cells were seeded in technical quadruplicate in a 96-well plate at 4000 cells/cm^2^. After 24 h in standard conditions, cells were treated for 72 h with ZF1 at three different concentrations (0.01, 10, and 20 μg/mL), or with a solvent (in equal amount), or were untreated (control cells) and were cultured at 37 °C in a complete medium with resazurin reagent (at the ratio of 10:1, respectively). In each experiment, negative (blue resazurin solution with medium) and positive (red totally-reduced resazurin with medium) controls in the absence of cells were added. Fluorescence (correlated to the presence of reduced resazurin as marker of cell metabolic activity over time) was measured after 4, 24, 48, and 72 h from the beginning of the treatment with the Wallac 1420 Victor2 multilabel counter (Perkin Elmer, Waltham, MA, USA) at an emission wavelength of 590 nm and an excitation wavelength of 560 nm. The number of viable cells correlating with the magnitude of dye reduction was expressed as a percentage of resazurin reduction according to this formula: (FI 590 of test agent–FI 590 of negative control)/(FI 590 of 100% reduced of resazurin–FI 590 negative control) × 100, where FI means fluorescence intensity.

Then, the same experiments were extended to hMSCs isolated from four sources (hASCs, hDP-MSCs, hBM-MSCs, and hWJ-MSCs), and ZF1 at the concentration of 20 μg/mL was used. Each treatment was performed in quadruplicate and the whole experiment was repeated with cells obtained from three different subjects (culture passages spanning from the 5th to the 7th).

### 4.6. Senescence-Associated β-Galactosidase Staining

Cell staining was performed using a SA β-gal kit (Cell Signaling, Danvers, MA, USA). Briefly, all investigated hMSCs were seeded at culture passages between 5th and 7th in 6-well plates (Falcon BD) at the density of 2000 cells/cm^2^. After 24 h in standard conditions, cells were treated for 72 h with ZF1 or with a solvent as a control. The preliminary experiments were conducted by using three ZF1 different concentrations (0.01, 10, and 20 μg/mL) in hASCs (*n* = 3). Then the experiments were extended to hMSCs isolated from four sources (hASCs, hDP-MSCs, hBM-MSCs, and hWJ-MSCs) using ZF1 only at the concentration of 20 μg/mL for 72 h and at the different density of 1770 cells/cm^2^ for hWJ-MSCs. Each treatment was performed in duplicate and the whole experiment was repeated with cells obtained from three different subjects. Cells were fixed and processed according to the manufacturer’s instructions. The number of positive (blue) and negative (not colored) cells was counted in each sample in at least three random fields under the microscope (at 200× magnification and bright field illumination) and the percentage of SA β-gal-positive cells was calculated as the number of positive cells divided by the total number of cells counted multiplied by 100 [71,72].

### 4.7. Adipogenic Differentiation

The hASCs at culture passages between 5th and 7th (*n* = 3) were tested for their ability to differentiate into adipogenic lineage. The hASCs were cultured on 24-well plates at the density of 15,000 cells/well. After 24 h in standard conditions, cells were treated for 72 h with ZF1 at three different concentrations (0.01, 10, and 20 μg/mL), or with a solvent as a control. Each treatment (ZF1 at different doses and solvent) was performed in six wells. At the end of the treatment, 4 wells were incubated with adipogenic medium (hMSC adipogenic differentiation medium, Lonza, Walkersville, MD, USA), which was changed twice a week for two weeks. The other two wells were not induced with adipogenic medium but cultured in a standard medium. At the end of the two weeks, differentiation was assessed using Oil Red O staining, as previously described [73]. In particular, cells were fixed in 10% formalin at room temperature for 15 min, washed in distilled water and incubated for 15 min with Oil Red O solution. Then, the cell monolayer was washed three times with demineralized H_2_O. Finally, Oil Red O was extracted by incubation with isopropanol for 10 min in moderate agitation. For each well, the dye was aliquoted and transferred in triplicate to a 96-well plate prior to reading absorbance at 495 nm using a spectrophotometer (Victor 2, Perkin Elmer Wallac, Milan, Italy).

Furthermore, the adipogenic differentiation assay was extended to hMSCs isolated from four sources (hASCs, hDP-MSCs, hBM-MSCs, and hWJ-MSCs) at the 5th culture passage. A ZF1 concentration of 20 μg/mL was used, and for each investigated hMSC type a technical triplicate was performed (*n* = 3).

### 4.8. RNA Extraction and RT-PCR

The hMSCs obtained from the investigated sources, were seeded in T25 flasks at the density of 3500 cells/cm^2^ (culture passages 5th–7th) and incubated in standard conditions for 24 h before treatments. The hASCs were treated for 72 h with 0.01, 10, and 20 μg/mL ZF1, or with the solvent as a control in a preliminary experiment (technical triplicate), and subsequently, each type of MSCs was treated for 72 h with 20 μg/mL ZF1, or with the solvent as a control. For each type of MSCs, the whole experiment was repeated in biological triplicate. Total RNA was extracted from all investigated cell lineages using the RNeasy mini kit (QIAGEN, Valencia, CA, USA) following the manufacturer’s instructions. The genomic DNA contamination was removed by digestion with RNase-free deoxyribonuclease I (RNase-free DNase set, QIAGEN). The reverse transcription of the extracted RNA was performed as previously described except for the temperature of the reaction that was 37 °C instead of 42 °C [74]. The success of the reaction was verified by the amplification of the human *Glyceraldehyde 3-phosphate dehydrogenase* (*GAPDH*) gene, using specific primers (forward sequence: 5′-GAAATCCCATCACCATCTTCCAG-3′ and reverse sequence 5′-GCTACACTGAGCACCAGGTGGTCTCCT-3′). The *GAPDH* amplification was performed as previously described [75], for 25 cycles instead of 45. The amplicon detection was performed by gel electrophoresis as previously described [25].

### 4.9. Real-Time PCR

A relative quantitative real-time PCR (qPCR) was performed in a Bio-Rad CFX96 real-time thermal cycler (Bio-Rad Laboratories, Hercules, CA, USA) as previously described [76]. Briefly, 25 ng of cDNA were amplified using the SsoAdvanced Universal SYBR Green Supermix (Bio-Rad Laboratories) in technical triplicate for every cDNA sample. Primers for *TERT* were purchased from Invitrogen (forward sequence: 5′-AAGTTCCTGCACTGGCTGATG-3′ and reverse sequence 5′-GCTTTGCAACTTGCTCCAGAC-3′) (Invitrogen, Carlsbad, CA, USA) and were used as 0.2 μM each in the qPCR reactions. Specific primers for *BMI1*, *TP53*, *CDKN1A (p21),* and *CDKN2A (p16)* (unique assay ID: qHsaCED0046537, qHsaCID0013658, qHsaCID0014498 and qHsaCED0056722, respectively, Bio-Rad Laboratories) were designed by Bio-Rad and used following the manufacturer’s instructions (20×, Bio-Rad Laboratories).

Relative gene expression was determined using CFX Manager Software version 3.1 (Bio-Rad Laboratories) with the “delta-delta CT method” [77] and *hypoxanthine phosphoribosyl transferase 1* (*HPRT1*), *TATA box binding protein* (TBP), *GAPDH* (20×, unique assay ID: qHsaCID0016375, qHsaCID0007122, qHsaCED0038674, respectively, Bio-Rad Laboratories) were used as reference genes. The investigated mRNA levels in hASCs, hDP-MSCs, hBM-MSCs, and hWJ-MSCs treated with ZF1 20 μg/mL were expressed as fold of change (2^−ΔΔCt^), relative to mRNA levels evaluated in respective hMSC lines treated with a solvent as a control. A preliminary gene expression analysis with qPCR was conducted to exclude effects of the treatment with solvent. Gene expression analysis was performed in biological triplicate for each cell type.

### 4.10. Protein Extraction and Western Blot

All investigated hMSCs were seeded at culture passages between 5th and 7th in T25 flasks (Falcon BD) at the density of 3500 cells/cm^2^. After 24 h in standard conditions, cells were treated for 72 h with ZF1 20 μg/mL or with a solvent as a control.

At the end of the treatment, cells were scraped from the flasks and pelleted at 1200 rpm for 5 min. Pellets were lysed with the mammalian protein extraction reagent (M-PER, Thermo-Fisher Scientific, Waltham, MA, USA) supplemented with 100× protease and phosphatase inhibitors (Sigma-Aldrich). Then, cell lysates were subjected to 3 cycles of 5 s of sonication followed by 2 min in ice and then centrifuged at the maximum speed to remove the cellular debris. The protein content of each sample was determined using a Bradford kit (VWR International, Radnor, Pennsylvania, USA).

Before electrophoresis, every sample was mixed with 4× Laemmly sample buffer (Bio-Rad) supplemented with a 10% of 2-mercaptoethanol (Sigma-Aldrich) and then boiled at 95 °C for 5 min.

An amount of 10 µg of proteins were loaded in Mini-PROTEAN TGX stain-free precast protein gels (Bio-Rad) and electrophoresis was performed in a 10× tris/glycine/SDS buffer (Bio-Rad) with the Mini-PROTEAN Tetra System (Bio-Rad). Gels and total protein amount were analyzed with ChemiDoc imaging system (Bio-Rad). Blotting was performed using a Trans-Blot Turbo Transfer Pack and a Trans-Blot Turbo Transfer System (Bio-Rad). After a blocking step of 1 h in a 3% solution of milk (Blotting Grade Blocking; Bio-Rad) in TBS-Tween 0.1%, membranes were incubated overnight at 4 °C with the p21 (Santa Cruz Biotechnologies, Dallas, Texas, USA) primary antibody used 1:1000 and 1 h at room temperature with the appropriate secondary antibody, diluted 1:5000 (Santa Cruz Biotechnologies). Protein detection was performed after 1 min of incubation with Clarity Max Western ECL substrate (Bio-Rad) and image acquired using ChemiDoc Touch Imaging System (Bio-Rad). The detected signal for each sample was normalized to the total protein detected in stain-free acquisitions. The Western blot analysis was performed in biological triplicate for each cell type.

### 4.11. Statistical Analysis

Data obtained from in vitro toxicology assay were investigated with ANOVA analysis followed by the Tukey HSD test. Data obtained from the SA β-gal assay were analyzed using the Student’s *t*-test in preliminary experiment and Z-test for proportional in the study of the four types of hMSCs treated with ZF1 20 μg/mL. Data obtained from the adipogenesis assay and the Western blot were analyzed using the Student’s *t*-test. Data obtained from qPCR were analyzed using the CFX Manager Software version 3.1 (Bio-Rad Laboratories) and the Student’s *t*-test. The results were considered statistically significant with a *p*-value < 0.05 and highly significant with a *p*-value < 0.01.

## Abbreviations

α-MEM	alfa-Minimal Essential Medium
BMI1	BMI1 proto-oncogene, polycomb ring finger
BM	bone marrow
CDKN1A (p21)	cyclin dependent kinase inhibitor 1A
CDKN2A (p16)	cyclin dependent kinase inhibitor 2A
c-Myc	v-myc avian myelocytomatosis viral oncogene homolog
CTR	control
DP	dental pulp
DMEM	Dulbecco’s Modified Eagle’s Medium
FBS	Fetal Bovine Serum
GAPDH	glyceraldehyde 3-phosphate dehydrogenase
h	hour
hpf	hours post fertilization
hMSCs	human mesenchymal stem cells
hASCs	human adipose tissue-derived stem cells
HPRT1	hypoxanthine phosphoribosyl transferase 1
iPS	induced pluripotent stem
LC-MS/MS	liquid chromatography-tandem mass spectrometry
OCT-4	POU domain class 5 homeobox 1 (POU5F1), alias Oct-4
qPCR	quantitative relative real-time PCR
SA β-gal	Senescence-Associated β-Galactosidase
SDS-PAGE	Sodium Dodecyl Sulphate-Polyacrylamide Gel Electrophoresis
SOLV	solvent
TBP	TATA box binding protein
TERT	telomerase reverse transcriptase
TP53	tumor protein p53
WJ	Wharton’s Jelly
ZF1	Zebrafish extract
